# Burden of childhood-onset arthritis

**DOI:** 10.1186/1546-0096-8-20

**Published:** 2010-07-08

**Authors:** Lakshmi N Moorthy, Margaret GE Peterson, Afton L Hassett, Thomas JA Lehman

**Affiliations:** 1 Robert Wood Johnson Medical School-UMDNJ, Dept. of Pediatrics Room 1361, 89 French Street, New Brunswick, NJ, USA; 2 Hospital For Special Surgery, 535 East 70th Street, New York, NY, USA; 3 University of Michigan Medical School, Chronic Pain & Fatigue Research Center, Dept of Anesthesiology, Ann Arbor, MI, USA; 4 Weill Medical College of Cornell University, New York, NY, USA

## Abstract

Juvenile arthritis comprises a variety of chronic inflammatory diseases causing erosive arthritis in children, often progressing to disability. These children experience functional impairment due to joint and back pain, heel pain, swelling of joints and morning stiffness, contractures, pain, and anterior uveitis leading to blindness. As children who have juvenile arthritis reach adulthood, they face possible continuing disease activity, medication-associated morbidity, and life-long disability and risk for emotional and social dysfunction. In this article we will review the burden of juvenile arthritis for the patient and society and focus on the following areas: patient disability; visual outcome; other medical complications; physical activity; impact on HRQOL; emotional impact; pain and coping; ambulatory visits, hospitalizations and mortality; economic impact; burden on caregivers; transition issues; educational occupational outcomes, and sexuality.

The extent of impact on the various aspects of the patients', families' and society's functioning is clear from the existing literature. Juvenile arthritis imposes a significant burden on different spheres of the patients', caregivers' and family's life. In addition, it imposes a societal burden of significant health care costs and utilization. Juvenile arthritis affects health-related quality of life, physical function and visual outcome of children and impacts functioning in school and home. Effective, well-designed and appropriately tailored interventions are required to improve transitioning to adult care, encourage future vocation/occupation, enhance school function and minimize burden on costs.

## Introduction

Juvenile Idiopathic Arthritis (JIA) is a chronic inflammatory disease causing erosive arthritis in children, often progressing to disability. JIA occurs in children of all races, with annual incidence ranging from 0.008-0.226 per 1000 children, and prevalence from 0.07-4.01 per 1000 children [1]. A 2007 Centers for Disease Control (CDC) study estimates that 294,000 U.S. children under age 18 (or 1 in 250 children) have been diagnosed with arthritis or another rheumatologic condition [2]. The national direct costs in 1989 are estimated at $285 million in the US for children with arthritis [3].

One of the most frequently occurring pediatric rheumatic diseases, JIA, impacts bio-psychosocial aspects of patients and families necessitating careful diagnosis and initiation of appropriate treatment. JIA is comprised of the following seven categories as per the ILAR (International League of Associations of Rheumatology) classification [4]:1) Oligoarticular onset JIA also termed as Pauciarticular onset JIA (PaJIA); 2) Polyarticular onset JIA (PoJIA RF+) with positive results on testing for Rheumatoid Factor (RF); 3) Polyarticular onset JIA (PoJIA RF-) with negative results on testing for RF; 4) Systemic arthritis (SoJIA); 5) Psoriatic arthritis JIA; 6) Enthesitis-related arthritis; and 7) Other arthritis undefined by above mentioned criteria [4]. Each subtype is associated with specific complications and disabilities. Early disease recognition and institution of appropriate therapy is critical to improve the clinical outcome.

As children who have JIA reach adulthood, they face possible continuing disease activity, medication-associated morbidity, life-long disability and risk for emotional and social dysfunction. Children with JIA with uveitis experience additional visual problems leading to disability. In this article we will review the burden of JIA for the patient and society focusing on the following areas: patient disability; visual outcome; other medical complications; physical activity; impact on health-related quality of life (HRQOL); emotional impact; pain and coping; ambulatory visits, hospitalizations and mortality; economic impact; burden on caregivers; transition issues; educational occupational outcomes; and sexuality. We will discuss the above topics under the three following headings: Physical Disability, Impact on HRQOL and Economic burden. Articles related to the above topics in the context of childhood arthritis were selected using MEDLINE search technique.

JIA comprises a heterogeneous group of diseases with distinct subtypes and all these subtypes have different phenotypes, course and prognoses. Although, we have included all the subtypes of JIA, we stress here that we need further studies in the following areas in each of the subtype. Further, not all the articles have exclusively used the ILAR system of classification. We have several studies on children with juvenile rheumatoid arthritis (JRA) cited throughout the manuscript.

## Physical Disability

### Disability

Several studies have elucidated the extent of disability associated with JIA. Solari et al explored the disease outcomes of a cross-sectional sample of 310 children with JIA for at least 5 years and found that about one-fifth had moderate to severe disability, 3.6% were in Steinbrocker class III-IV, and about 10% had major impairment in HRQOL [5]. One-third of the patients had damage in at least one joint or joint group and one-fourth showed extraarticular damage. Thirty-five percent of the 150 patients having a wrist radiograph had significant structural damage, while 9% had growth retardation [5]. Since a considerable proportion of the patients had active disease and attendant disability, impaired function, and damage, the cumulative burden will impose a significant load on health care resources.

Russo et al studied 47 patients with SOJIA with at least 2 years of disease and found that 43% patients exhibited damage (38% with articular and 19% with extraarticular damage) [6]. The chief determinants of damage were cervical spine arthritis and corticosteroid usage in the first 6 months of the disease course. Further, the damage scores correlated with number of joints with limited motion, and functional disability [6]. In another study of 89 JIA patients, almost two-thirds of patients had articular damage and one third had extra-articular damage [7]. Growth failure was the most common extra-articular manifestation. Juvenile arthritis damage index (JADI)-A (a measure or articular damage) correlated with the following variables: (a) radiological damage; (b) childhood health assessment questionnaire (CHAQ); (c) JADI-E (measure of extraarticular damage); (d) disease; and (e) loss of education years due to disease; and (f) measures of disease activity [7].

In a longitudinal analysis, 227 patients with JIA were given functional ability questionnaires to complete [8]. The total number of completed questionnaires was 1,356. The interval between first and last questionnaire administration was 949.7 patient years. Three longitudinal patterns were observed. First pattern was the absence of disability was found in 27.8% of patients and persistently moderate disability in 3.5%. Second pattern was the finding of a steady improvement (22.9%) or deterioration (5.7%) in disability over time. The third pattern was the finding of a fluctuating course of disability, with deterioration and improvement (40.1% of patients). The chief determinants of poor functional ability were younger age at disease onset and a greater restricted joint count. A wide within-patient and between-patient variability in the longitudinal course of functional disability was found. Children with early disease onset and a greater number of restricted joints had the highest risk of developing long-term physical disability [8].

A Mexican cross-sectional study examined the impact of disability on the quality of life in 32 Mexican adults with polyarticular course JIA (mean age 26 years) and juvenile ankylosing spondylitis (JAS) (mean age 27 years) [9]. A significant proportion of patients with JIA (65%) and JAS (50%) had Global Functional Status (GFS) class III/IV. Thirty-two percent of JIA patients had a score greater than 1.5 when assessed by the Health Assessment Questionnaire-disability index (HAQ-DI). Ninety-two percent of the JAS patients had a score greater than 5 when examined by the Bath Ankylosing Spondylitis Functional Index (BASFI). A higher BASFI score indicates worse functional ability. All patients had lower scores for all physical and mental subscales of the medical outcomes study 36-item short-form health survey (SF-36) when compared with Mexican population scores (p < 0.005). But health status did not differ between the patients with JIA and JAS. EQ-5D, a quality of life scale showed impairment in all dimensions for JIA and JAS patients. The primary determinant of quality of life (QOL) as measured by SF 36 questionnaire was the GFS score. These results suggest that functional status significantly impairs HRQOL in adults with JIA and JAS [9].

The above studies from 2007-2008 represent the transition period to biologic agents and several of these patients were probably on methotrexate and non-biologic agents. As biologic agents are gaining popularity, next decade may present different results such as lower level of disability and increased functional capacity.

### Visual outcome

Children with PaJIA, PoJIA, psoriatic arthritis and enthesitis-related arthritis are all at risk for uveitis with potential for visual loss. In order to examine the long-term outcome of JIA-associated uveitis, Ozdal et al studied 18 adults with JIA-associated uveitis with mean duration of 21 years [10]. The mean age of the patients at last examination was 30 years and the mean age at onset of arthritis and uveitis was 5 and 9 years respectively. All patients had eyes with at least one ocular complication [10]. Final visual acuity was impaired in 40%, poor in 20%, and completely lost in 10% of patients. Overall, 70% of the eyes were either visually handicapped or totally blind. Most eyes endured at least one surgical procedure. All patients were on topically and systemic NSAID; and 61% of patients were on an immunosuppressive agent [10].

Kump et al conducted a retrospective review and found that of 269 children with uveitic syndromes, 89 (33%) had JIA-associated uveitis, and it was bilateral in most (76 children) [11]. Mean age of onset of uveitis was about 6 years and mean follow up was about 3 years. Children with JIA-associated uveitis developed the following complications in the course of their disease: cataracts (64%); increased intraocular pressure (20%); developed band keratopathy (46%); and posterior synechiae (58%). The treatment regimen was as follows: single-/two-/three- or greater immunomodulating agents (73%); nonsteroidal anti-inflammatory agents alone or in combination with immunomodulating agents (40%); and topical and/or systemic steroids (21%). Using mixed-models linear regression, improvement in visual acuity was not found to be statistically significant [11].

A more recent retrospective chart review of a subset of 1,081 JIA patients under 18 years with at least one year follow-up at a single center revealed the following: 13% developed uveitis after a mean follow-up of 6 years; complications related to uveitis were observed in 37% of cases; and 15% with uveitis underwent a total of 62 ocular surgeries [12]. Good visual acuity was found in 91% of eyes; impaired visual acuity in 6 eyes (3%), and blindness in 10 eyes (6%). Children with JIA achieved good visual outcome despite uveitic complications [12]. Children with JIA with uveitis on topical steroids often require cataract surgery. While the surgery improves their outcomes significantly [13], it imposes a financial burden. Another recent long-term study of 55 patients with JIA-associated uveitis showed 42% of patients developed cataracts at 7 years and in 51% at 24 years [14]. Further, 5% developed glaucoma 7 years and 22% at 24 years [14]. After 24 years, almost one-half of the patients (27) still had active uveitis [14]. These studies reflect the blinding potential of JIA-associated uveitis. The complications of uveitis are significant including chronic medication use and physician appointments, surgery, and visual handicap, all of which result in impaired quality of life.

### Physical activity

Lelieveld et al found that total energy expenditure, activity-related energy expenditure, and physical activity levels were significantly lower in the 30 adolescents with JIA compared to 106 controls [15]. Adolescents with JIA spent a greater percentage of time in bed and less time on moderate to vigorous physical activity. Only 23% of the JIA patients met public health guidelines on physical activity compared with 66% in the control group [15].

## Other Related Medical Complications

### Podiatry

Foot problems are prevalent in patients with JIA (over 90%) [16] (reviewed in). In one study of 30 patients with JIA, 63% reported some foot impairment; 53% reported foot-related activity limitation; and participation restriction was endorsed by 60%. Ten children (one-third) sought podiatry care. Although immunomodulating and biologic therapies are increasingly used, foot-related impairment is significant and requires podiatry care [16].

### Gastrointestinal symptoms related to therapy

While performing the initial validation of Gastrointestinal Symptom Scale for Kids (GISSK) in children with juvenile rheumatoid arthritis (JRA), Brunner et al found that gastrointestinal (GI) symptoms were present in the majority of the patients with JRA (58%) [17]. Patients with moderate or severe GI symptoms requiring advanced therapies were associated with lower HRQOL [17].

### Amyloidosis

In a Finnish study, patient registers and amyloidosis biopsy files from 1976-2003 from the Center of inflammatory joint disorders were examined for amyloidosis in patients under age 19 years [18]. Additionally, medical records were reviewed, and patients were interviewed by telephone. Death certificates were reviewed to determine cause. Twenty-four patients under age 19 years with biopsy-proven amyloidosis were found. Authors found the 5-year survival rate of the series to be 87.5% (95% CI 75% to 100%), and 10-year survival to be 75% (95% CI 54% to 92%). Ten out of the 24 died during a mean follow-up of 15.4 years. The chief cause of death was related to JIA in 9 patients. Patients treated with corticosteroids alone after the diagnosis of amyloidosis had a higher mortality than those taking disease modifying anti-rheumatic drugs (p = 0.001). Of the 14 patients who were alive, 12 had normal renal function (3 of them had undergone renal transplantation), one had renal insufficiency, and one had proteinuria. All the 14 patients had completed at least the 9 years of mandatory education, and 4 had academic degrees. Two female patients had delivered healthy children. This Finnish study suggests that the outcome of JIA-associated amyloidosis is poor overall [18].

## Impact on HRQOL, Pain and Coping

### Impact on HRQOL

Several studies have examined the impact of JIA on HRQOL. Patients with JIA and reactive arthritis had lower HRQOL compared to normative data [19]. Children had lower HRQOL in the domain of self-esteem; adolescents had lower HRQOL in the areas of physical well-being and total QOL. About one-fifth of the cohort seemed to have significant behavior problems, mostly social isolation and depression and/or anxiety. Functional impairment impacted HRQOL and behavioral issues. The chief determinants for poor HRQOL were functional limitations, social isolation and depression and/or anxiety [19]. Shaw et al examined the HRQOL in 308 adolescents and found that HRQOL of adolescents with JIA was less than optimal, particularly in the domains of gross motor and systemic functioning [20]. In another cohort of 88 JIA patients, variables "limitation of mobility" and "pain" showed the robust association with HRQOL, implying that HRQOL is directly influenced by the consequences of the illness [21]. Sixty children with JIA and/or their parents/proxies participated in a recent study. Subjects with active disease had lower scores on the Pediatric Quality of Life Inventory (PedsQL™) Generic Core Scales and the PedsQL™ Rheumatology Module than those with inactive disease. Participants also reported more fatigue compared to controls regardless of disease activity status [22].

Another study assessing 80 subjects with JIA reported that children with polyarticular and systemic subtypes had worse fine and gross motor function, greater psychosocial impact and greater number of symptoms compared to patients with persistent oligoarticular type [23]. HRQOL was decreased in adolescents and worsened with delay in diagnosis. The decrease of HRQOL was linked to disease activity, disability index (measured by CHAQ) and presence of hip involvement. This study implies that JIA has substantial adverse impact on HRQOL of patients, principally adolescents with polyarticular and systemic subtypes. The chief predictors of poor HRQOL are disease duration, disability score (CHAQ) and pain [23].

In a multinational, cross-sectional study, HRQOL was assessed in 3,324 JIA patients using the Child Health Questionnaire (CHQ) and compared to HRQOL of 3,315 healthy children [24]. At the time of the study visit, JIA patients were younger (mean age 10 years) than the healthy controls (mean age 11 years). There were more girls in the JIA group compared to controls; however, there was no difference between the physical and psychosocial scores between the healthy males and females. The mean physical and psychosocial summary scores of the CHQ were significantly lower in patients with JIA than in healthy children, with the physical well-being domain being most affected. Patients with persistent oligoarthritis had better HRQOL compared to other subtypes; and HRQOL was similar in patients with systemic arthritis, polyarthritis, and extended oligoarthritis. Poor physical function (CHAQ score > 1) and increased pain intensity (pain intensity rating > 3.4 cm) on a 10-cm visual analog scale were the strongest predictors of poorer HRQOL in the physical and psychosocial domains, respectively. Clearly, HRQOL of children with JIA is greatly impacted in the area of physical function. Physical well-being was influenced by the extent of functional disability, and pain intensity affected psychosocial health [24]. These studies confirm that these patients have significantly impaired HRQOL compared to healthy children and the physical function domain is primarily involved.

### Impact on mental health

While impact of JIA on physical function and global HRQOL is well characterized, there are fewer studies that address the emotional impact of JIA. Mullick et al evaluated 40 subjects (24 boys) aged 10-18 years with JIA for at least one year and 40- age and sex-matched healthy controls were included as controls [25]. The frequency of psychiatric disorders (based on ICD-10 clinical diagnoses of multiaxial classification of child and adolescent psychiatric disorders) was significantly higher in JIA (35%) than in the control group (12.5%). The increased length of illness was linked with a higher percentage of cases with psychiatric disorders. In the JIA group, the frequencies of the various diagnoses were as follows: depressive disorder (15%), somatoform disorder (12.5%), adjustment disorder (5%), and mixed anxiety and depressive disorder (2.5%). In addition, the JIA group with psychiatric problems had substantially higher stressors, perceived difficulties, distress, social impairment, and burden for caregivers were reported in the JIA group with psychiatric morbidity. The presence of psychiatric disorders was related to considerable difficulties with learning, peer relationships, and leisure activities. This study suggests that early recognition of psychiatric illness and management might improve the outcome in children with JIA [25].

Vuorimaa et al examined the relationship between arthritis in 145 children and self-efficacy, trait-anxiety, depression, clinical state of the disease (pain, disability, number of somatic complaints and active joints) and age of the child [26]. Interestingly, clinical classification of disease activity and severity was not directly linked to depression and trait-anxiety in children with JIA. It was self-efficacy that corresponded with less pain and somatic complaints [26].

Ding et al investigated the psychological functioning of 60 children with polyarticular joint disease and its association with disease activity and disability. The authors found that all facets of psychological function (depression, anxiety and behavior) correlated moderately with physical function. Poor psychological outcome was linked with more severe physical disability, but not with the extent of disease activity [27]. Clearly, more studies are needed to determine the extent and pattern of emotional factors on the symptoms, functioning and HRQOL of patients with JIA.

### Pain and coping

Thastum et al examined whether the pain-specific beliefs and coping strategies of JIA patients independently predicted their reported pain [28]. Results from this study supported a model of pain experience in patients with JIA where psychological factors are strongly influential. Their findings suggested that behavioral interventions may be highly relevant in patients who experience pain that appears out of proportion to the disease activity [28].

Sawyer et al examined the relationship between HRQOL, the experience of pain and pain coping strategies in 59 children (8-18 years) with JIA and their parents [29]. Parents stated significantly lower HRQOL scores compared to children on five of the eight PedsQL™ scales rating children's HRQOL. The authors found a notable inverse linkage between pain levels and the PedsQL™ scores assessing children's physical, emotional and social functioning for both children and parents. They also found a significant inverse relationship between scores on numerous pain coping scales and scores on the PedsQL™ scales. The authors concluded that intensity and pain coping strategies have a significant and independent relationship with several domains of HRQOL. Further, parents and children have differing views of the nature of these relationships, highlighting the need for obtaining child and parent reports [29].

### Burden on caregivers

Parents often have to miss days of work to care for their sick children. Parents have to bring children to their physician's regular appointments, and additional urgent and emergency room visits during disease flares or infections (since they are often immunocompromised). In addition, parents have to schedule appointments to obtain laboratory tests and imaging studies. Parents have to frequently refill one or more medications. These activities can result in significant emotional, financial and logistical burden for the parents. Bruns et al examined the QOL and disease burden of primary caregivers of 70 patients with JIA [30]. They used the CHAQ, SF-36, and the psychiatric screening questionnaire (SRQ-20). Burden of disease on the caregivers was measured by the caregiver burden scale (CB Scale). Majority of the caregivers were married mothers with an average age of 37 years; most of them had low education and socioeconomic status. Psychoemotional disorders were identified in one-third of the caregivers. Items on pain and mental health from the SF-36 questionnaire were the most impacted. The CB Scale correlated with the SRQ-20, number of limited joints, number of visits, family income, and mental health, emotional aspects, social aspects, vitality and general health state of the SF-36. SRQ-20 was the most critical determinant of CB Scale and of the domains of pain and mental health of the SF-36 questionnaire. Patients' emotional status (rather than the physical status) likely contributes a greater load on the caregivers [30].

### Transition issues

In order to delineate the transitional care workload of a multicentre cohort of adolescents with juvenile idiopathic arthritis (JIA) including disease, self-advocacy and vocational issued, Shaw et al collected data using questionnaires completed by clinicians, patients and parents in 10 UK pediatric rheumatology centers [31]. Patients with a confirmed diagnosis of JIA for at least 6 months and an age of 11, 14 or 17 yrs were included. 308 adolescents with JIA and 303 parents/guardians participated and of these, 19.5% had persistent oligoarthritis. Despite their upcoming transfer to adult care, the following transitional issues were prevalent in the 17-yr-olds:

Over half the patients were accompanied by their parents during their rheumatology appointment; one-fifth of the patients were not self-medicating; two-thirds had not had intra-articular injections under local anesthetic; and one-seventh had not received counseling related to their future careers.

Over half of the 17 yr-olds had moderate to severe functional disability. Sixty-eight percent were still on disease-modifying anti-rheumatic drugs and, experienced increased pain compared to the younger patients. The transitional care workload appears to be substantial for both pediatric and adult rheumatologist in terms of improving self-efficacy, disease-factors and adjustment to future careers [31].

### Educational and occupational outcomes

Gerhardt et al investigated the educational and occupational outcomes among young adults with JIA and peers during their transition from adolescence to adulthood [32]. Authors enrolled families when children with JIA were 8-14 years old and each child was age-, sex-, and race-matched with a classmate. There were 45 JIA patients (about 12 years after diagnosis), 46 peers and parents who completed questionnaires after the adolescent's 18^th ^birthday. Surprisingly, young adults with JIA and peers were comparable in terms of family background, scholastic and occupational self-concept, and academic competence. The percentage of high school graduates and those working, those planning for further studies or seeking employment was equivalent in both groups [32]. Disease subtype, severity at presentation and time elapsed were not associated with indices of educational and occupational accomplishment. This study indicates that in spite of JIA and the different associated challenges, young adults are similar to their peers as they transition to adulthood [32].

### Sexuality

There is limited data on sexuality of adolescents and young adults with JIA. A questionnaire was developed by de Avila Lima Souza et al for the evaluation of sexuality of male patients with JIA [33]. The authors studied a cohort of male patients with rheumatoid factor (RF)-negative polyarticular JIA. Thirty-two male patients with ages 16-26 and disease duration 13-20 years had mean HAQ score 0.1-2.1. Practice of masturbation, regular sexual intercourse, preserved desire and satisfaction were not significantly different between patients and controls. However, joint pain during intercourse was more frequent in patients (48% vs. 3% in controls; p < 0.001). Increased mean HAQ score was found in the 12 patients with joint pain during intercourse compared to the 13 patients without joint pain [33]. Further studies are required to explore details of sexual function in a larger sample of both male and female adolescents and young adults with different disease subtypes.

## Economic Burden

### Financial costs

Arthritis and related conditions, such as juvenile arthritis, cost the U.S. economy nearly $128 billion per year in medical care and indirect expenses, including lost wages and productivity [34]. Great financial burden is imposed on families of patients with JIA and may be attributed to the following: expensive biologic agents; frequent subspecialty outpatient visits; periodic laboratory and imaging tests; frequent visits to the pediatrician for increased URI, etc. due to immunosuppression; physical therapy; inpatient infusions and hospitalizations; and missed work days for the parents. The process of diagnosis and treatment can be very expensive [35]. Minden et al measured direct costs (healthcare and non-healthcare costs) and indirect costs (productivity loss due to sick leave and work disability) in 215 JIA patients in adulthood [36]. These patients were assessed on an average of 17 years after disease onset using clinical evaluation, a structured interview, and two self completion questionnaires [36]. The authors assessed annual direct costs based on the reported use of healthcare services and resources, using average unit prices. Indirect costs were determined from the number of work days missed-that is, using the human capital approach. The mean total cost of late JIA was estimated to be 3500 Euros per patient and year, of which the direct cost contributed a large percentage. Ninety percent of the cost was attributable to patients with still active disease (55%). These patients incurred a mean total cost of 5700 Euros per patient year, and among them those under rheumatologic care incurred a cost of 9300 Euros. Independent contributors to increased costing patients with active JIA included the following variables: belonging to a certain JIA subgroup, functional disability, and receipt of specialized care. Highest mean total costs were found in patients with active seropositive polyarthritis (17,000 Euros) and extended oligoarthritis (11,000 Euros), while the lowest were found in active enthesitis related arthritis (1,500 Euros) and persistent oligoarthritis (2,700 Euros) [36]. Authors concluded that the estimated one-year costs in are considerable and variable among the various JIA subgroups [36].

Allaire et al collected data on direct costs, family costs and community (extra school) costs on 120 families with children with JRA in New England, and published the results in 1992 [3]. The mean yearly direct cost per child was $7,905 (inpatient: $1,717; outpatient: $5,700; and nonmedical: $488). Family costs averaged $1,524/year (out of pocket medical and nonmedical: $1,196; lost salary: $328), which corresponded to 5% of mean family income. The mean extra school cost was $1,449/9 months. This study shows that the economic impacts of JRA is significant [3].

Given the fluctuating nature of the disease, the need for treatment escalation, physical therapy, screening for ophthalmologic complications and surgery, cumulative costs need to be determined. Especially with the advent of biologic therapies, treatment strategies in JIA should be analyzed for their long-term cost effectiveness [36].

### Ambulatory health care visits, hospitalizations, and mortality

The National Ambulatory Medical Care Survey and National Hospital Ambulatory Medical Care Survey found that among children < 17 years in 1997-98, there was an average of 1.3 million AORC-related ambulatory care visits per year related to arthritis and other rheumatic conditions (AORC) [37]. Hootman et al reported that pediatric arthritis-related visits were more likely to be made by girls (67%), whites (82%), non-Hispanics (66%) and children were aged 12-17 years (59%). Most visits occurred in physician offices (75%) compared to outpatient departments (18%) and emergency department (7%). The main three diseases seen were: soft tissue disorders excluding back (41%; 513,000), unspecified joint pain/effusion (31%; 387,000), and rheumatoid arthritis (10%; 122,000). Among physician office visits, 41% saw family practice/general practitioners/internal medicine; 33% saw rheumatologists/orthopedists/neurologists; and 26% saw pediatricians [37].

Sacks et al estimated the prevalence of and the annual number of ambulatory health care visits (physician, outpatient, emergency) for children with significant pediatric arthritis and other rheumatologic conditions (SPARC) using data from the 2001-2004 National Ambulatory Medical Care Survey and 2001-2004 National Hospital Ambulatory Medical Care Survey [2]. Visit estimates were converted into prevalence estimates using data on the number of prior annual visits per patient. Synthetic estimates for states were extrapolated using national rates. The average annualized number of children with SPARC was 294,000 (95% confidence interval [95% CI] 188,000-400,000); and ambulatory health-care visits were 827,000 (95% CI 609,000-1,044,000) [2]. Study data also show that children diagnosed with arthritis and other rheumatologic conditions had an average of 83,000 emergency department room visits annually [2]. With codes for AORC as defined in adults, in 1997, among children < 14 years, there were 21,000 hospitalizations with chief diagnosis of AORC (rate = 3.5/10,000) and 33,000 hospitalizations with any reference to AORC (rate = 9.2/10,000) out of 2,266,000 childhood hospitalizations (0.9%-1.45% of all hospitalizations)[38]. Undoubtedly, the number of arthritis-related health care visits is significant and impacts the families' HRQOL and burdens the pediatric health care system. Detailed tracking of such data for different subtypes of JIA is important over the long-term [2].

Sacks et al reviewed multiple cause of death tapes from the National Center for Health Statistics (NCHS) from 1979 to 1998. The authors found that the crude death rate from AORC was 2.46 per 100,000 in 1979, and by 1998, it was 3.48. The three categories of AORC that accounted for almost 80% of deaths were diffuse connective tissue diseases (34%), other specified rheumatic conditions (23%), and rheumatoid arthritis (22%) [39].

Using AORC codes, the Centers for Disease Control and Prevention's (CDC) NCHS death data revealed that about 1,000 children younger than 15 years of age died from arthritis and other rheumatic conditions in the 20 years from 1979-1998 (average = 50 deaths/year). The juvenile AORC death rate fell 25% during the 20-year period from 1.2 per million to 0.9 per million (average = 1 death per million children per year) [39] (study quoted in the CDC website-[40]).

Hashkes et al described mortality rates, causes of death, and potential mortality risk factors in pediatric rheumatic diseases in the US using the Indianapolis Pediatric Rheumatology Disease Registry, which included 49,023 patients from 62 centers who were newly diagnosed between 1992 and 2001[41]. Authors confirmed 110 deaths among 48,885 patients (0.23%). Patients had been followed up for a mean ± SD of 7.9 ± 2.7 years. The standardized mortality ratios (SMR) of the entire cohort was significantly decreased (0.65 [95% CI 0.53-0.78]), with differences in patients followed up for > or = 9 years. The SMR was significantly greater for systemic lupus erythematosus (3.06 [95% CI 1.78-4.90]) and dermatomyositis (2.64 [95% CI 0.86-6.17]) but not for systemic juvenile rheumatoid arthritis (1.8 [95% CI 0.66-3.92]). Causes of death were related to the rheumatic diagnosis (including complications) in 39 patients (35%), treatment complications in 11 (10%), non-natural causes in 25 (23%), background disease in 23 (21%), and were unknown in 12 patients (11%). Rheumatic diagnoses, age at diagnosis, sex, and early use of systemic steroids and methotrexate were significantly associated with the risk of death. Their findings showed that the overall mortality rate for pediatric rheumatic diseases was not increased. Even for the diseases and conditions associated with increased mortality, mortality rates were significantly lower than those reported in previous studies [41]. Current data are required for patients on biologic treatments. Further, in order to elucidate the impact on cost, hospitalizations, ambulatory care and emergency room visits codes specific for childhood arthritis should be used and specified for particular disease and subtype.

## Conclusion

Juvenile arthritis imposes a significant burden on different spheres of the patients', caregivers' and family's life. In addition, it imposes a societal burden of significant health care costs and utilization. Juvenile arthritis affects health-related quality of life, physical function and visual outcome of children and impacts functioning in school and home. The extent of impact on the various aspects of the patients', families' and society's functioning is clear from the existing literature. Effective, well-designed and appropriately tailored interventions are required to minimize the burden on costs; enhance transitioning to adult care, improve school function and future vocation/occupation and overall the long-term outcome of patients.

In the next two decades, some of the aspects of disease course may significantly change with the increasing use of biologic agents for treatment of patients with juvenile arthritis. The impact of juvenile arthritis on the various aspects of the patients', families' and societies' functioning would require re-examination at that time. Creating and sharing world-wide prospective registries on all patients with childhood arthritis would enable us to garner data on the burden of the disease subtypes, health outcomes, and safety of medications, as we transition to the era of biologic medications.

We emphasize again that the subtypes of JIA together are very different diseases and associated with unique complications. Despite that, we can still accurately acknowledge that the various subtypes of JIA impose a major physical, psychosocial and economic burden on the patient, family and society; and currently we are in need for appropriate interventions.

## Competing interests

The authors declare that they have no competing interests.

## Authors' contributions

LNM (submitting author) primarily created and drafted the manuscript and revised it based on co-authors' suggestions. MGEP, ALH and TJAL have been involved in revising it critically for important intellectual content and have given final approval of the version to be published.
